# Estimating interactions in individual participant data meta-analysis: a comparison of methods in practice

**DOI:** 10.1186/s13643-022-02086-0

**Published:** 2022-10-05

**Authors:** Ruth Walker, Lesley Stewart, Mark Simmonds

**Affiliations:** Centre for Reviews and Dissemination, University of York, Heslington, York, YO10 5DD UK

**Keywords:** Individual patient data, Meta-analysis, One-stage, Two-stage, Subgroups

## Abstract

Medical interventions may be more effective in some types of individuals than others and identifying characteristics that modify the effectiveness of an intervention is a cornerstone of precision or stratified medicine. The opportunity for detailed examination of treatment-covariate interactions can be an important driver for undertaking an individual participant data (IPD) meta-analysis, rather than a meta-analysis using aggregate data. A number of recent modelling approaches are available. We apply these methods to the Perinatal Antiplatelet Review of International Studies (PARIS) Collaboration IPD dataset and compare estimates between them. We discuss the practical implications of applying these methods, which may be of interest to aid meta-analysists in the use of these, often complex models.

Models compared included the two-stage meta-analysis of interaction terms and one-stage models which fit multiple random effects and separate within and between trial information. Models were fitted for nine covariates and five binary outcomes and results compared.

Interaction terms produced by the methods were generally consistent. We show that where data are sparse and there is low heterogeneity in the covariate distributions across trials, the meta-analysis of interactions may produce unstable estimates and have issues with convergence. In this IPD dataset, varying assumptions by using multiple random effects in one-stage models or using only within trial information made little difference to the estimates of treatment-covariate interaction. Method choice will depend on datasets characteristics and individual preference.

**Supplementary Information:**

The online version contains supplementary material available at 10.1186/s13643-022-02086-0.

## Introduction

Systematic reviews and meta-analyses are widely used within healthcare to combine relevant data from individual clinical studies. They form an integral part of evidence-based medicine and aim to provide robust evidence to inform policy and clinical practice. Systematic review with individual participant data (IPD) meta-analysis has been referred to as a ‘gold standard’ approach to evidence synthesis [1]. In addition to producing more reliable and robust summary effect estimates, an important reason for collecting IPD is to understand if particular types of patients benefit more or less from a treatment. This is achieved through investigating whether important clinical covariates, such as age, sex or previous medical history alter treatment effectiveness, also termed “effect modification” [2].

The ability to conduct this type of analysis is important within healthcare as it underpins more personalised approaches to clinical intervention by informing the types of individual to whom intervention is offered [3]. Tailoring healthcare to individuals also has potential to save resources, for example, restricting use to those individuals where it is most effective or cost effective or avoiding the burden of treatment in individuals who derive little or no benefit.

### Methods of estimating treatment—covariate interaction

Exploring effect modification involves the estimation of treatment-covariate interaction through the inclusion of interaction parameters within meta-analytic models [4]. This can be done through the use of meta-regression based on published results/aggregate data but may lack power and is prone to aggregation bias and confounding [4]. In IPD synthesis, treatment-covariate interaction can be estimated using either ‘two-stage’ or ‘one-stage’ meta-analysis. Two-stage methods produce estimates of interaction within each trial which are then combined using conventional meta-analysis and either fixed or random effects. One-stage methods combine data from all relevant trials in a single analysis using regression modelling. The approach does not analyse data as if they come from a single ‘mega-trial’, but maintains the differentiation between trials and accounts for the clustering of patients within the trials [5]. Due to this, the approach is also sometimes termed a ‘stratified analysis’ [3].

There has been growing consideration of methods for the estimation of treatment-covariate interaction, with debate in the literature as to how they differ, and which is method best to use and when. Simmonds and Higgins [4] first compared meta-regression, the two-stage meta-analysis of interactions (MAOI) and a one-stage method and determined the power of the methods to detect treatment-covariate interactions. Authors concluded that the power of meta-regression depends on the variation in the mean covariate values across studies and that statistical power may be lacking when studies are few. The method is also prone to aggregation bias and confounding. The power of the MAOI model depends on the variation of covariates within each study, whilst the one-stage model always has at least equal or greater statistical power compared to meta-regression and the MAOI model [4].

Since then, methods have advanced and it has become possible to fit more complex one-stage models, including those with multiple random effects. There is now suggestion these may be better than models that use a single random effect [6]. Furthermore, as one-stage models do not automatically avoid aggregation bias when estimating treatment-covariate interactions, there has been consideration of methods that separate within-trial and across-trial information. It has been recommended that one-stage IPD-MA consider only within-trial estimates to avoid biased results driven by aggregation bias [7–9].

Simulation studies have compared these methods for estimating treatment-covariate interaction. Da Costa and Sutton [10] used simulated IPD to compare six one-stage models, which varied in whether or not they accounted for variation in between-trial interaction effects. Those accounting for this variation were less prone to bias and had more accurate standard errors [10]. These results are generalisable to trials using continuous outcomes. In this context, the research highlights the importance of separating out with and between trial effects results. Another simulation study conducted by Kontopantelis [11] used continuous outcome data to compare one-stage models that used fixed or random treatment effects and the two-stage MAOI. The study found that one-stage models consistently outperformed the two-stage model when estimating interactions, but considered only one-stage models that combined within and across-study information [11], and so did not account for aggregation bias.

Belias et al. [12] used simulated binary IPD to compare four models of estimating treatment-covariate interaction. These were meta-regression, by trial subgroup analysis, the MAOI model, a one-stage model (referred to as a ‘naïve one-stage IPD-MA’) that used only a random treatment effect and a one-stage model that centred effect modifiers by their mean in each trial to account for potential aggregation bias. Both the one-stage models were found to have greater power than other models, were unaffected by heterogeneity levels and showed increased power in scenarios with aggregation bias. The MAOI model had less power but was unaffected by heterogeneity levels and showed increased power in scenarios with aggregation bias. Conversely, the by-trial subgroup analysis lost power in scenarios where between-study heterogeneity was increased. Meta-regression showed poor power in all scenarios [12].

Using simulated data with often extreme scenarios, to compare methods, may not accurately reflect how the methods will perform in practice and rather demonstrate how they are expected to perform in theory. Comparison of methods for estimating treatment-covariate interaction using real world datasets is limited. Stewart et al. [3] used obstetric data, collected by the Perinatal Antiplatelet Review of International Studies (PARIS) Collaboration to compare one-stage models with fixed vs. random treatment effects and interaction effects and one model with separated within and across-trial treatment-covariate interactions. One-stage and two-stage models were used to estimate treatment-covariate interactions for pre-eclampsia (the main outcome). Authors concluded that models produced similar results but discussed advantages of the one-stage model, over the two-stage model, including greater flexibility to explore model structure [3].

In this paper, we use the same PARIS dataset to consider and compare additional one-stage and two-stage models to estimate interactions, building on the work by Stewart et al. [3]. We also consider more recent modelling approaches which fit multiple random effects and separate within and between trial information. We analyse five main outcomes and nine covariates in the PARIS dataset and examine whether different methods produced different results. We also explore and discuss the practical implications of applying these methods to a large IPD dataset, which will be of interest to statisticians and using the methods in practice.

## Methods

The PARIS dataset contains IPD from 31 randomised controlled trials with health outcomes for 32,217 women and their 32,819 babies. It explores the efficacy of antiplatelet therapy in the prevention of pre-eclampsia and its complications. Women were randomised to receive an antiplatelet therapy or a placebo [13].

We used PARIS data for five main outcomes: pre-eclampsia, pre-term birth prior to 34 weeks, small for gestational age infant, foetal or neonatal death and the composite outcome, pregnancy with a serious adverse outcome (SAO). Treatment-covariate analysis for these outcomes considered all available binary covariates in the PARIS dataset.

- 1st pregnancy—family history of hypertensive disorder of pregnancy (HPD)
- 2nd pregnancy—previous history of HPD
- 1st pregnancy—any high risk factor*
- 2nd pregnancy—any high risk factor*
- Pre-existing renal disease
- Pre-existing hypertension
- Pre-existing diabetes
- Previous infant SGA
- Multiple pregnancy

*A ‘high risk’ pregnancy was defined as a current pregnancy with any of the following: maternal autoimmune disease, renal disease, diabetes or chronic hypertension, or with abnormal uterine artery Doppler flow, multiple pregnancy, family history of HDP, or an unspecified risk factor as defined within the trial. Otherwise, a previous pregnancy with a history of any of the following: gestational hypertension, pre-eclampsia, eclampsia, foetal or neonatal death each of which were collected and included in the dataset as individual variables [13].

The number of women with and without an event (aggregate counts across all trials) for each outcome and covariate of interest, by treatment allocation are presented in Supplementary Material Table 1. Analysis originally conducted by Askie et al. [13] used participant-level subgroup analysis [13], an approach commonly in early IPD meta-analyses which produces subgroup-level effect estimates within study before pooling these estimates in meta-analyses using conventional techniques. Pooled estimates are compared using a test for interaction such as the Cochrans’ *Q* test.

We investigated the use of more recent methods and compared the estimates of treatment-covariate interaction coefficients produced by the two-stage MAOI model and five one-stage models including those with common or random interaction effects [6] and one model that uses only within-trial information on the treatment-covariate interaction. These methods are described in detail in Table 1. The interaction estimates produced by the models, indicate the extent to which one subgroup is likely to benefit more or less from a treatment.

**Table 1:** Model characteristics for one two-stage and five one-stage models for estimating treatment covariate interaction in an individual participant meta-analysis

Model	Equation	Modelling assumptions
***Two stage model:*** In the first stage, maximum likelihood regression model is used within each trial (Simmonds and Higgins 2007 [4]), including a treatment effect and a treatment-covariate interaction term. In the second stage, the interaction effect estimates from each trial (${\hat{\upgamma}}_i$) are combined using conventional meta-analysis techniques (in this case, the inverse-variance meta-analysis using the DerSimonian-Laird random effect method), producing a summary treatment-covariate interaction estimate.		
***Meta-analysis of interactions*** (Simmonds and Higgins 2007 [4])	*g*(*y*_*ij*_) = Φ_*i*_ + θ_*i*_*x*_*ij*_ + *μ*_*i*_*z*_*ij*_ + *γ*_*i*_*x*_*ij*_*z*_*ij*_	● The studies are estimating a different, yet related interaction effects.
***One-stage models:*** A one-stage maximum likelihood regression model includes both a treatment effect and a treatment-covariate interaction term, with data from all studies in the same model. The common effect version of the model is as equation for meta-analysis of interactions, except now the parameters are assumed common across all studies. A separate intercept term (Φ_𝑖_) retains distinctions between studies, avoiding the assumption that data arise from one ‘mega trial’		
***Common interaction effect: model*** (Tuner *et al.* 2000 [14])	$${\displaystyle \begin{array}{c}\textrm{g}\left({y}_{ij}\right)={\Phi}_i+\left(\theta +{u}_i\right)\ {x}_{ij}+\mu {z}_{ij}+\gamma {x}_{ij}{z}_{ij}\\ {}{u}_i\sim N\left(0,{\tau}^2\right)\end{array}}$$	● The true effect of the treatment is allowed to vary between studies. ● The true effect of the interaction is assumed common between studies.
***Common interaction effect: model 2*** (Jackson et al. 2018 [6])	$${\displaystyle \begin{array}{c}\textrm{g}\left({y}_{ij}\right)=\left(\Phi +{v}_i\right)+\left(\theta +{u}_i\right)\ {x}_{ij}+\mu {z}_{ij}+\gamma {x}_{ij}{z}_{ij}\\ {}\left(\begin{array}{c}{u}_i\\ {}{v}_i\end{array}\right)\sim N\left(\left(\begin{array}{c}0\\ {}0\end{array}\right),\left(\begin{array}{cc}{\tau}_{\theta}^2& \lambda \\ {}\lambda & {\tau}_{\phi}^2\end{array}\right)\right)\end{array}}$$	● The true effect of the treatment is allowed to vary between studies. ● The true effect of the interaction is common between studies. ● The random effects for the trial and treatment are correlated.
***Common interaction effect: model 3*** (Jackson et al. 2018 [6])	$${\displaystyle \begin{array}{c}\textrm{g}\left({y}_{ij}\right)=\left(\Phi +{v}_i\right)+\left(\theta +{u}_i\right)\ {x}_{ij}+\mu {z}_{ij}+\gamma {x}_{ij}{z}_{ij}\\ {}\left(\begin{array}{c}{u}_i\\ {}{v}_i\end{array}\right)\sim N\left(\left(\begin{array}{c}0\\ {}0\end{array}\right),\left(\begin{array}{cc}{\tau}_{\theta}^2& \lambda \ast \\ {}\lambda \ast & {\tau}_{\phi}^2\end{array}\right)\right)\end{array}}$$ *λ = 0	● The true effect of the treatment is allowed to vary between studies. ● The true effect of the interaction is common between studies. ● The random effects for the trial and treatment are uncorrelated.
***Random interaction:***	$${\displaystyle \begin{array}{c}\textrm{g}\left({y}_{ij}\right)=\left(\Phi +{v}_i\right)+\left(\theta +{u}_i\right)\ {x}_{ij}+\mu {z}_{ij}+\left(\gamma +{w}_i\right){x}_{ij}{z}_{ij}\\ {}\left(\begin{array}{c}{u}_i\\ {}{v}_i\\ {}{w}_i\end{array}\right)\sim N\left(\left(\begin{array}{c}0\\ {}0\\ {}0\end{array}\right),\left(\begin{array}{ccc}{\tau}_{\theta}^2& 0& 0\\ {}0& {\tau}_{\phi}^2& 0\\ {}0& 0& {\tau}_{\gamma}^2\end{array}\right)\right)\end{array}}$$	● The true effect of the treatment is allowed to vary between studies. ● The true effect of the interaction is allowed to vary between studies. ● The random effects for the trial, treatment and interaction are uncorrelated.
***Within study model***	$${\displaystyle \begin{array}{c}\textrm{g}\left({y}_{ij}\right)=\left(\Phi +{v}_i\right)+\left(\theta +{u}_i\right)\ {x}_{ij}+\mu {z}_{ij}+\xi {x}_{ij}\left({z}_{ij}-{\overline{z}}_i\right)+\upeta {\overline{z}}_i\\ {}\left(\begin{array}{c}{u}_i\\ {}{v}_i\end{array}\right)\sim N\left(\left(\begin{array}{c}0\\ {}0\end{array}\right),\left(\begin{array}{cc}{\tau}_{\theta}^2& 0\\ {}0& {\tau}_{\phi}^2\end{array}\right)\right)\end{array}}$$ ${\overline{z}}_i$ is the average covariate value in trial i, so *ξ* is the parameter for the within-trial interaction.	● The effect of the treatment and covariates are assumed common between studies. ● Only the within-study information on the treatment-covariate interaction is used, avoiding the assumption that the observed across-study relationships do reflect the individual-level relationships within trials.

𝑖 indicates the trial (1 to *k*), and *j* participants within each trial (1 to *n*_*i*_) *y*_*ij*_ is the participant outcome with an identity for continuous outcomes or a logit link (odds ratios) or log link (risk ratio) for dichotomous outcomes; *x*_*ij*_ usually takes the value one for treatment group and zero for control group; *z*_*ij*_ is value of the covariate for each participant. Hence, Φ_𝑖_ is the intercept term, *θ*_*i*_ is the treatment effect, *μ*_*i*_ the covariate effect, and *γ*_*i*_ is the treatment-covariate interaction (the parameter of interest)

We consider only models that include a single covariate. One-stage models may be extended to include multiple covariates, which would correct for any correlation between covariates. However, as the PARIS dataset reports different covariates in different trials, it was not possible to consider a model that include all covariates for these analyses.

Also, not considered are meta-regression and by-trial subgroup analyses, as these analyses are only useful for study level covariates or characteristics common across the trial, and therefore, hold no substantive benefit above conducting a convention meta-analysis using aggregate data [12, 15]. The two-stage participant-level subgroup analysis is also not considered here as the method does not produce a treatment-covariate interaction coefficient. Furthermore, comparisons using this method are made between subgroups within studies and so the method sometimes lacks the statistical power to be able to detect treatment-covariate interactions [3, 4].

Finally, this research has been carried out to assess the impact of modelling options and not to determine clinical outcomes. As such, we have not calculated statistical power for these interaction analyses.

## Results

**Fig. 1:**
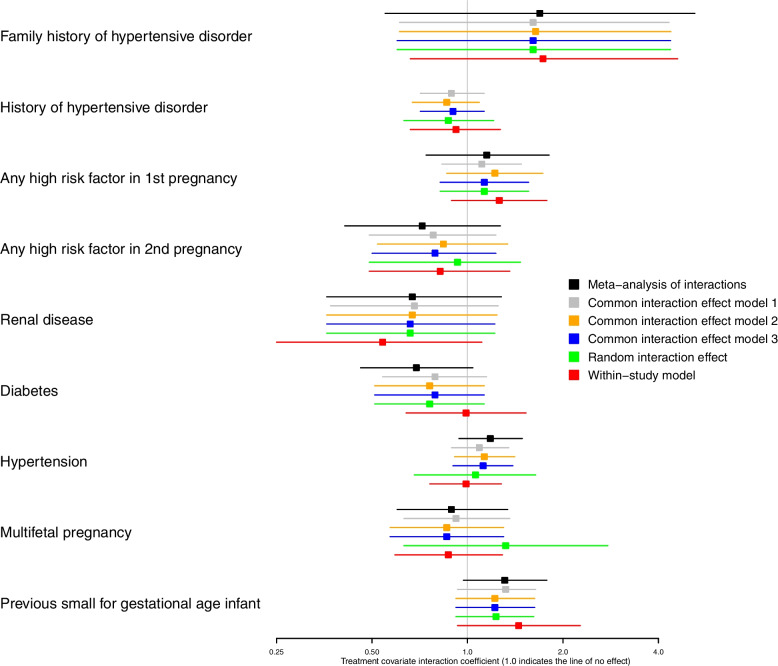
Estimates of treatment-covariate interaction for the outcome pre-eclampsia

**Fig. 2:**
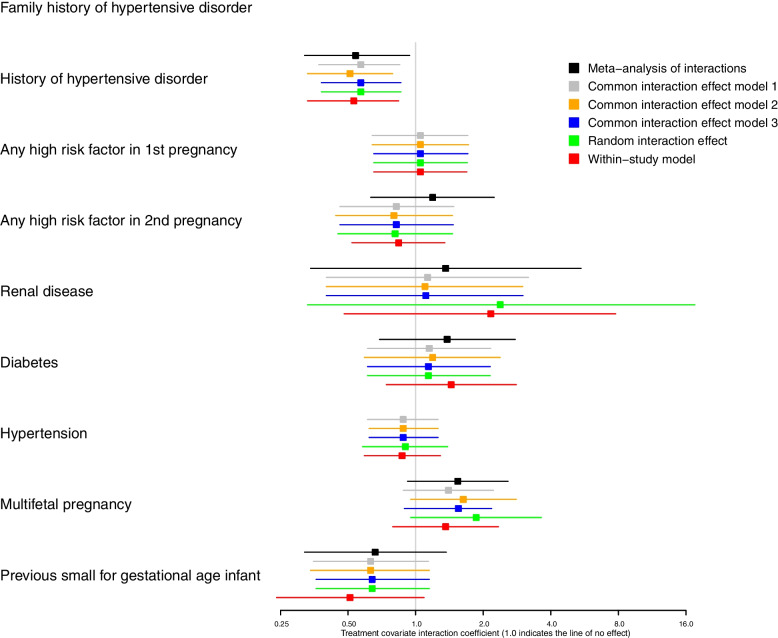
Estimates of treatment-covariate interaction for the outcome fetal or neonatal death

Fifty-four outcome-covariate combinations were considered equating to 270 analyses (Figs. 1 and 2, Supplementary Material Figure 1, 2, 3 and Tables 2–6). Figure 1 contains estimates of treatment-covariate interaction coefficients for the main outcome pre-eclampsia, produced by the MAOI model and the five one-stage models. Figure 2 contains interaction estimates for a rarer outcome, foetal or neonatal death. Here, no model coverage for covariate ‘family history of hypertensive disorder’ for the outcome foetal or neonatal death, due to a lack of data (Fig. 2). In general, analyses demonstrate that the six methods produced consistent estimates throughout.

Overall, few analyses showed any clear evidence of treatment-covariate interaction, generally having wide confidence intervals. The only exceptions were history of hypertensive disorder for several outcomes including foetal or neonatal death (Fig. 2, Supplementary Material Figure 1, 2) and for multifetal pregnancy, small for gestational age infant and pre-term birth < 34 weeks (Supplementary Material Figures 2, 3). Here, interaction estimates were consistent with confidence intervals generally excluding the line of no effect.

In some cases, the choice of method altered the statistical significance of the interaction estimate, for example, for history of HPD-pregnancy with covariate pregnancy with a serious adverse outcome, history of HPD-pregnancy with covariate pre-term birth < 34 weeks and multifetal pregnancy with covariate SGA (Supplementary Material Figures 1, 2, 3). However, in all cases, the maximum difference in point estimate between methods was generally small ≤ 0.08, and the confidence intervals were close to the line of no effect.

There were occasions where interaction estimates between methods differed more substantially, for example, for the outcome foetal or neonatal death and covariate renal disease (Fig. 2). For this analysis, the greatest difference in the treatment-covariate interaction coefficient between methods was 1.06, between the common interaction effect: model two (OR 1.1 (95% CI 0.40–3.00)) and the aggregation bias: within study model (OR 2.16 (95% CI 0.48–7.78)). Estimates for the latter model were more uncertain (Fig. 2). Differences in interaction estimates produced by the random interaction effect model were also noted for several outcomes with the covariate multifetal pregnancy (Figs. 1 and 2, Supplementary Material Figures 1 and 3).

The aggregation bias: within-study model and the MAOI model more commonly produced estimates that differed from the one-stage models, which ‘amalgamate’ within trial and across-trial information. For example, those produced for the covariate diabetes (Figs. 1 and 2). These differences, however, were not always consistent between the MAOI and aggregation bias: within study model (Figs. 1 and 2) and estimates generally had wider confidence intervals than those produce by the one-stage common interaction effect models.

When producing estimates using the MAOI, within-study regression models would not converge within trials where there were low to zero events or where all participants within a trial all had the same event status for a given covariate. These trials dropped out of the analysis and therefore, in some cases, the number of trials contributing to the meta-analysis of interactions and to one-stage models differed. This was sometimes coupled with observed differences in estimates, for example, with the outcome baby death and covariate renal disease (Fig. 2), where only two studies converged within the MAIO analyses compared to five in the one-stage model. In eight instances, the one-stage models produced estimates of treatment-covariate interaction, whereas the meta-analysis of interaction failed to converge across any trial and produced no estimate (Supplementary Material Tables 2–5).

## Discussion

Previous comparison of one-stage and two-stage methods of meta-analysis for estimating treatment-covariate interaction have used simulated data [10, 11]. We have compared these methods using IPD to understand how the methods perform in practice. We considered five main outcomes and nine covariates in the PARIS dataset. For the majority of analyses, the MAOI model and five one-stage model produced very similar estimates of treatment-covariate interaction coefficients, aligning with findings of previous research [3, 4].

Generally, the choice of analytic method had very limited impact on the estimate of treatment-covariate interaction and very rarely produced differences such as, altering the statistical significance of an estimate, which would lead to differing conclusions.

In some analyses, the MAOI model and the within-study model produced point estimates that differed substantially from those produced by other methods (Fig. 2). These models synthesise only the within-study information to examine treatment-covariate relationship and avoid making inference about individual relationships within trials, based on the observed across-study relationships (trials may differ in ways other than the covariate under examination). As such, both of the methods avoid aggregation bias.

Most one-stage models do not automatically avoid aggregation bias when estimating treatment-covariate interactions. As such, comparing of the results from one-stage models with those from the MAOI model and the within study model might reveal erroneous estimates produced by the one-stage models methods that aggregate both within-trial and across-trial information. However, in our analyses, the MAOI model and the within study model did not produce differences with the one-stage models consistently, suggesting it may not be a real effect driving these differences. As differences were more common for outcomes where events were rare, it is more likely that the observed differences in results are attributable to limited data where the meta-analysis of interactions model lacks power [4].

The MAOI model and the within-study model, should, in theory, be the most unbiased methods as they avoid the use of across-study information, which may lead to erroneous inference, should differences in mean covariate values exist across studies [7–9]. In analyses like ours, where trial populations are sufficiently similar to one another that aggregation bias does not pose a great issue, there may be a case to include across-trial information, which would improve the power of the interaction estimate compared to within-trial information alone [8].

Pragmatically, issues arose when implementing the MAOI method, with regression models within study failing to converge. Applying this method to large IPD datasets where outcome events are rare and there are few participants with particular clinical covariates may be difficult in practice. This is because participants with and without both the covariate and the outcome are needed to produce a within-study estimate using the MAOI method. Zero cell counts for binary outcomes can be overcome by using continuity correction, where 0.5 is added to cells in the available 2 × 2 table. However, this approach should be applied with caution as it has previously been shown to influence the magnitude of the effect estimates and their variances [5].

For one-stage models methods that aggregate both within-trial and across-trial information, our analyses generally showed little difference between estimates. Previous work has suggested that using models with multiple random effects produce more accurate estimates than those with single random effects [6]. We noted differences in the interaction estimate produced by the random interaction model for analyses that included the covariate multifetal pregnancy. Here, few trials had enough data to estimate the interaction (multifetal pregnancies were generally low across trials < 50). Adding a random effect on the interaction needs some within-trial interaction data to estimate the heterogeneity, and so, in these analyses, the models became unstable and the estimates uncertain.

One-stage and two-stage models examining treatment-covariate interactions are each associated with advantages and disadvantages. For example, the meta-analysis of interactions method comes with the ability to produce forest plots enabling easier visualisation of the contribution that each study makes to the summary effect estimate. This may be useful during preliminary investigations. Software environments such as R with increasingly available pre-written code means that the level of statistical expertise required to implement the methods is now similar between two-stage and one-stage models, despite some previous suggestion that one-stage models are computationally more complex [3].

Different models make different assumptions about the effect of parameters on the interaction estimate, and although this did not greatly alter estimates in this work, when considering which method to use, it is important to consider the appropriateness of assumption in the context of your data, alongside the practicality of applying the models. As we have demonstrated, it is important to consider ‘covariate heterogeneity’ or the heterogeneity in the covariate distributions across studies. To do this, we recommend cross-tabulating the number of events occurring in covariate groups for outcomes of interest prior to data-analyses. If covariate heterogeneity is low, then meta-analysis of interactions models may fail to converge, and estimates produced by such models may be unstable. In this case, a one-stage model would be the preferable choice. Should covariate heterogeneity be high, then the meta-analysis of interactions will likely be comparable to the one-stage model [4] and wider factors may be considered when choosing between methods.

## Limitations

Our analyses used a 1/0 coding for the treatment variable. Other authors have suggested that a + 0.5/− 0.5 coding would be preferable to ensure a common variance for treatment and control groups and improve maximum likelihood estimation [6, 16], particularly in random effect models where trials are few and the estimation of correlation between two random effects is problematic [14]. Alternatively a ‘study-specific centering’ (coding 1/0 minus the study-specific proportion of participants in the treatment group) could have been applied that can reduce the downward bias of between-study variance when using maximum likelihood estimation [16].

To further understand which methods perform best in practice, a wider range of real-world applications including those which consider continuous covariates is needed.

## Conclusion

In this empirical example, varying assumptions within the one-stage model made little difference when estimating treatment-covariate interaction. As trial populations were sufficiently similar, aggregation bias did not pose a great issue, and as such, applying models that separate within and between study information did not hold substantive value. As the methods are capable of producing differing results in some circumstances, it is important to pre-specify method choice in a study protocol, to avoid post hoc testing which attempts to achieve statistical significance.

## Supplementary Information

**Additional file 1: Supplementary Material.** R code used in analysis of IPD.

**Additional file 2: Supplementary Material Table 1.** Number of women with and without an event (aggregate counts across all trials) for each outcome and covariate of interest, by treatment allocation. **Supplementary Material Table 2.** Treatment-covariate interaction coefficients estimated by six methods meta-analysis for the outcome pre-eclampsia and nine covariates. **Supplementary Material Table 3.** Treatment-covariate interaction coefficients estimated by six methods meta-analysis for the outcome fetal or neonatal death and nine covariates. **Supplementary Material Table 4.** Treatment-covariate interaction coefficients estimated by six methods meta-analysis for the outcome pregnancy with a serious adverse outcome and nine covariates. **Supplementary Material Table 5.** Treatment-covariate interaction coefficients estimated by six methods meta-analysis for the outcome preterm birth <34 weeks and nine covariates. **Supplementary Material Table 6.** Treatment-covariate interaction coefficients estimated by six methods meta-analysis for the outcome small for gestational age infant and nine covariates.

**Additional file 3: Supplementary Material Figure 1.** Estimates of treatment-covariate interaction for the outcome serious adverse outcome.

**Additional file 4: Supplementary Material Figure 2.** Estimates of treatment-covariate interaction for the outcome preterm birth before 34 weeks.

**Additional file 5: Supplementary Material Figure 3.** Estimates of treatment-covariate interaction for the outcome serious adverse outcome small for.

## Data Availability

Data sharing is not applicable to this article as no new data were created or analysed in this study. Access to the PARIS dataset was granted by the PARIS collaboration who hold the dataset.

## Abbreviations

HPD: Hypertensive disorder of pregnancy
IPD: Individual participant data
MA: Meta-analysis
MAOI: Meta-analysis of interactions
PARIS: Perinatal Antiplatelet Review of International Studies
SAO: Serious adverse outcome
