# Supplementary material for: Estimating interactions in individual participant data meta-analysis: a comparison of methods in practice
Source: Syst Rev. 2022 Oct 5;11:211. doi: 10.1186/s13643-022-02086-0 (PMC9535994; doi:10.1186/s13643-022-02086-0)
Supplement: Supplementary file 1 — Additional file 1: Supplementary Material. R code used in analysis of IPD. [file 13643_2022_2086_MOESM1_ESM.docx]

**R computer code:**

***Estimating treatment-covariate interaction using meta-analysis of interactions***

# Run regression model in each trial

glmer (Outcome ~ Covariate* Treatment, family= bionomial (link “log”))

# Combine summary interaction effect estimates (γ̂𝑖) using conventional meta-analysis techniques

***Estimating treatment-covariate interaction using a one stage meta-analysis with random treatment effect***

glmer (Outcome ~ Trial + Covariate* Treatment + (Treatmentn-1|Trial), family= bionomial (link “log”))

***Estimating treatment-covariate interaction using a one stage meta-analysis with correlated trial treatment random effects***

glmer(outcome ~ factor(arm)*covar + (1+arm|trial),family=binomial)

***Estimating treatment-covariate interaction using a one stage meta-analysis with uncorrelated trial treatment random effects***

glmer(outcome ~ factor(arm)*covar + (1|trial) + (arm-1|trial),family=binomial)

***Estimating treatment-covariate interaction using a one stage meta-analysis with random treatment and random interaction effects***

glmer(outcome ~ factor(arm)*covar + (1|trial) + (arm-1|trial) + (arm:covar-arm-1|trial),family=binomial)

***Estimating treatment-covariate interaction using a one-stage meta-analysis that separates the within-study information on the treatment-covariate interaction from the between-study information^9^.***

glmer(outcome ~ factor(arm)*(covar.within+covar.between) + (1+arm|trial), family=binomial)
