# Supplementary material for: Estimating interactions in individual participant data meta-analysis: a comparison of methods in practice
Source: Syst Rev. 2022 Oct 5;11:211. doi: 10.1186/s13643-022-02086-0 (PMC9535994; doi:10.1186/s13643-022-02086-0)
Supplement: Supplementary file 2 — Additional file 2: Supplementary Material Table 1. Number of women with and without an event (aggregate counts across all trials) for each outcome and covariate of interest, by treatment allocation. Supplementary Material Table 2. Treatment-covariate interaction coefficients estimated by six methods meta-analysis for the outcome pre-eclampsia and nine covariates. Supplementary Material Table 3. Treatment-covariate interaction coefficients estimated by six methods meta-analysis for the outcome fetal or neonatal death and nine covariates. Supplementary Material Table 4. Treatment-covariate interaction coefficients estimated by six methods meta-analysis for the outcome pregnancy with a serious adverse outcome and nine covariates. Supplementary Material Table 5. Treatment-covariate interaction coefficients estimated by six methods meta-analysis for the outcome preterm birth <34 weeks and nine covariates. Supplementary Material Table 6. Treatment-covariate interaction coefficients estimated by six methods meta-analysis for the outcome small for gestational age infant and nine covariates. [file 13643_2022_2086_MOESM2_ESM.docx]

*Table 1.* *Number of women with and without an event (aggregate counts across all trials) for each outcome and covariate of interest, by treatment allocation.*

| Outcomes | | Pre-eclampsia | | Pre-term birth prior to 34 weeks | | Small for gestational age infant | | Fetal or neonatal death | | Pregnancy with a serious adverse outcome. | |
| --- | --- | --- | --- | --- | --- | --- | --- | --- | --- | --- | --- |
| Covariate | | Treatment (n/N) | Control (n/N) | Treatment (n/N) | Control (n/N) | Treatment (n/N) | Control (n/N) | Treatment (n/N) | Control (n/N) | Treatment (n/N) | Control (n/N) |
| 1^st^ pregnancy– Family history of HDP | Yes | 12 / 62 | 8 / 52 | 7 / 62 | 6 / 52 | 7 / 62 | 8 / 52 | 0 / 17 | 1 / 23 | 9/ 59 | 11/ 54 |
|  | No | 470 / 8463 | 531 / 8409 | 475 / 8428 | 488 / 8401 | 279 / 5061 | 289 / 5060 | 245 / 8488 | 254 / 8463 | 847/ 5617 | 813/ 5563 |
| 2^nd^ pregnancy – Previous history of HDP | Yes | 499 / 3116 | 479 / 2991 | 190 / 3189 | 247 / 3027 | 106 / 2909 | 120 / 2781 | 65 / 2855 | 97 / 2673 | 289 / 2596 | 361 / 2468 |
|  | No | 289 / 3799 | 302 / 3849 | 340 / 3927 | 367 / 3952 | 194 / 2707 | 206 / 2738 | 174 / 3980 | 171 / 4043 | 521 / 2370 | 559 / 2448 |
| 1^st^ pregnancy – any high risk factor* | Yes | 195 / 1194 | 212 / 1176 | 153 / 1196 | 145 / 1177 | 142 / 1281 | 150 / 1265 | 153 / 1196 | 145 / 1177 | 259 / 1073 | 260 / 1062 |
|  | No | 287 / 7335 | 327 / 7288 | 329 / 7297 | 349 / 7277 | 144 / 3839 | 147 / 3843 | 329 / 7297 | 349 / 7277 | 563 / 4549 | 598 / 4609 |
| 2^nd^ pregnancy – any high risk factor* | Yes | 659 / 5375 | 720 / 5281 | 448 / 5507 | 515 / 5354 | 274 / 4883 | 286 / 4756 | 187 / 5330 | 213 / 5180 | 689 / 4532 | 768 / 4441 |
|  | No | 79 / 1545 | 79 / 1556 | 82 / 1621 | 99 / 1625 | 20 / 435 | 42 / 434 | 52 / 1483 | 55 / 1499 | 121 / 434 | 152 / 476 |
| Pre-existing renal disease | Yes | 21 / 240 | 31 / 210 | 22 / 240 | 25 / 200 | 7 / 209 | 10 / 180 | 7 / 218 | 7 / 182 | 30 / 192 | 33 / 172 |
|  | No | 814 / 11131 | 896 / 11072 | 639 / 10916 | 700 / 10889 | 271 / 6223 | 332 / 6215 | 312 / 10449 | 319 / 10442 | 954 / 6033 | 1072 / 6043 |
| Pre-existing hypertension | Yes | 293 / 1678 | 295 / 1625 | 163 / 1659 | 184 / 1597 | 119 / 1634 | 127 / 1573 | 78 / 1523 | 90 / 1504 | 266 / 1527 | 287 / 1491 |
|  | No | 849 / 11641 | 958 / 11603 | 750 / 11434 | 815 / 11431 | 357 / 6952 | 390 / 6953 | 358 / 11408 | 378 / 11391 | 1117 / 6526 | 1237 / 6552 |
| Pre-existing diabetes | Yes | 60 / 439 | 82 / 466 | 47 / 433 | 40 / 462 | 16 / 367 | 16 / 392 | 7 / 218 | 7 / 182 | 65/ 365 | 65 / 391 |
|  | No | 1053 / 12707 | 1138 / 2601 | 853 / 12496 | 941 / 12412 | 442 / 8168 | 489 / 8093 | 312 /10449 | 319 /10442 | 1287 / 7626 | 1427 / 7597 |
| Previous infant SGA | Yes | 187 / 1635 | 160 / 1491 | 124 / 1645 | 130 / 1490 | 83 / 1607 | 93 / 1464 | 31 / 1459 | 43 / 1315 | 197 /1416 | 219 /1304 |
|  | No | 308 / 3419 | 370 / 3498 | 182 / 3618 | 219 / 3627 | 71 / 2419 | 93 / 2488 | 109 / 3412 | 99 / 3411 | 272 / 2222 | 321 / 2286 |
| Multiple pregnancy | Yes | 57 / 544 | 71 / 577 | 163 / 612 | 155 / 645 | 95 / 1024 | 83 / 1084 | 78 / 1211 | 70 / 1286 | 220 / 519 | 204 / 556 |
|  | No | 1114 / 14325 | 1206 / 14187 | 820 / 14595 | 911 / 14426 | 433 / 9216 | 467 / 9096 | 392 / 13855 | 424 / 13675 | 1283 / 7926 | 1421 / 9159 |

HDP: hypertensive disorder of pregnancy * A ‘high risk’ pregnancy was defined as a current pregnancy with any of the following: maternal autoimmune disease, renal disease, diabetes or chronic hypertension, or with abnormal uterine artery Doppler flow, multiple pregnancy, family history of HDP, or an unspecified risk factor as defined within the trial. Otherwise, a previous pregnancy with a history of any of the following: gestational hypertension, pre-eclampsia, eclampsia, foetal or neonatal death each of which were collected and included in the dataset as individual variables ^13^.

Table 2. Treatment-covariate interaction coefficients estimated by six methods meta-analysis for the outcome pre-eclampsia and nine covariates.

| **Covariate**  **Covariate** | **Meta-analysis of interactions** | **Common interaction effect model one** | **Common interaction effect model two** | **Common interaction effect model three** | **Random interaction effect** | **Within-study model** |
| --- | --- | --- | --- | --- | --- | --- |
|  | OR (95% CI) | OR (95% CI) | OR (95% CI) | OR (95% CI) | OR (95% CI) | OR (95% CI) |
| Family history of hypertensive disorder | 1.69(0.55 - 5.21) | 1.61 (0.61 - 4.32) | 1.64 (0.61 - 4.38) | 1.61 (0.6 - 4.37) | 1.61 (0.6 - 4.37) | 1.73 (0.66 - 4.6) |
| History of hypertensive disorder | Did not converge | 0.89 (0.71 - 1.13) | 0.86 (0.67 - 1.09) | 0.9 (0.71 - 1.13) | 0.87 (0.63 - 1.21) | 0.92 (0.66 - 1.27) |
| Any high-risk factor-1^st^ pregnancy | 1.15(0.74 - 1.81) | 1.11 (0.83 - 1.48) | 1.22 (0.86 - 1.73) | 1.13 (0.82 - 1.56) | 1.13 (0.82 - 1.56) | 1.26 (0.89 - 1.78) |
| Any high-risk factor-2ndpregnancy | 0.72(0.41 - 1.27) | 0.78 (0.49 - 1.23) | 0.84 (0.52 - 1.34) | 0.79 (0.5 - 1.23) | 0.93 (0.49 - 1.47) | 0.82 (0.49 - 1.36) |
| Renal Disease | 0.67(0.36 - 1.28) | 0.68 (0.37 - 1.25) | 0.67 (0.36 - 1.24) | 0.66 (0.36 - 1.22) | 0.66 (0.36 - 1.22) | 0.54 (0.25 - 1.11) |
| Diabetes | 0.69(0.46 - 1.04) | 0.79 (0.54 - 1.15) | 0.76 (0.51 - 1.13) | 0.79 (0.51 - 1.13) | 0.76 (0.51 - 1.13) | 0.99 (0.64 - 1.53) |
| Chronic hypertension | 1.18(0.94 - 1.49) | 1.09 (0.89 - 1.35) | 1.13 (0.91 - 1.41) | 1.12 (0.9 - 1.39) | 1.06 (0.68 - 1.64) | 0.99 (0.76 - 1.28) |
| Multifetal Pregnancy | 0.89(0.6 - 1.34) | 0.92 (0.63 - 1.36) | 0.86 (0.57 - 1.3) | 0.86 (0.57 - 1.3) | 1.32 (0.63 - 2.77) | 0.87 (0.59 - 1.29) |
| Previous small for gestational age infant | 1.31(0.97 - 1.78) | 1.32 (0.93 - 1.64) | 1.22 (0.92 - 1.63) | 1.22 (0.92 - 1.63) | 1.23 (0.92 - 1.62) | 1.45(0.93-2.27) |

Table 3. Treatment-covariate interaction coefficients estimated by six methods meta-analysis for the outcome fetal or neonatal death and nine covariates

| **Covariate**  **Covariate** | **Meta-analysis of interactions** | **Common interaction effect model one** | **Common interaction effect model two** | **Common interaction effect model three** | **Random interaction effect** | **Within-study model** |
| --- | --- | --- | --- | --- | --- | --- |
|  | OR (95% CI) | OR (95% CI) | OR (95% CI) | OR (95% CI) | OR (95% CI) | OR (95% CI) |
| Family history of hypertensive disorder | Did not converge | Did not converge | Did not converge | Did not converge | Did not converge | Did not converge |
| History of hypertensive disorder | 0.54 (0.32 - 0.94) | 0.57 (0.37 - 0.85) | 0.51 (0.33 - 0.79) | 0.57 (0.38 - 0.86) | 0.57 (0.38 - 0.86) | 0.53 (0.33 - 0.84) |
| Any high-risk factor-1^st^ pregnancy | Did not converge | 1.05 (0.64 - 1.71) | 1.05 (0.64 - 1.72) | 1.05 (0.65 - 1.71) | 1.05 (0.65 - 1.7) | 1.05 (0.65 - 1.69) |
| Any high-risk factor-2ndpregnancy | 1.19 (0.63 - 2.24) | 0.82 (0.46 - 1.48) | 0.8 (0.44 - 1.46) | 0.82 (0.46 - 1.47) | 0.81 (0.45 - 1.46) | 0.84 (0.52 - 1.35) |
| Renal Disease | 1.36 (0.34 - 5.46) | 1.13 (0.4 - 3.18) | 1.1 (0.4 - 3) | 1.11 (0.4 - 3.01) | 2.38 (0.33 - 17.52) | 2.16 (0.48 - 7.78) |
| Diabetes | 1.38 (0.69 - 2.78) | 1.15 (0.61 - 2.16) | 1.19 (0.59 - 2.38) | 1.14 (0.61 - 2.15) | 1.14 (0.61 - 2.15) | 1.44 (0.74 - 2.81) |
| Chronic hypertension | Did not converge | 0.88 (0.61 - 1.26) | 0.88 (0.62 - 1.26) | 0.88 (0.62 - 1.26) | 0.9 (0.58 - 1.39) | 0.87 (0.59 - 1.29) |
| Multifetal Pregnancy | 1.54 (0.92 - 2.58) | 1.4 (0.88 - 2.22) | 1.63 (0.95 - 2.81) | 1.55 (0.89 - 2.18) | 1.86 (0.95 - 3.63) | 1.36 (0.79 - 2.34) |
| Previous small for gestational age infant | 0.66 (0.32 - 1.37) | 0.63 (0.35 - 1.14) | 0.63 (0.34 - 1.15) | 0.64 (0.36 - 1.15) | 0.64 (0.36 - 1.15) | 0.51 (0.24-1.09) |

Table 4.Treatment-covariate interaction coefficients estimated by six methods meta-analysis for the outcome pregnancy with a serious adverse outcome and nine covariates

| **Covariate**  **Covariate** | **Meta-analysis of interactions** | **Common interaction effect model one** | **Common interaction effect model two** | **Common interaction effect model three** | **Random interaction effect** | **Within-study model** |
| --- | --- | --- | --- | --- | --- | --- |
|  | OR (95% CI) | OR (95% CI) | OR (95% CI) | OR (95% CI) | OR (95% CI) | OR (95% CI) |
| Family history of hypertensive disorder | Did not converge | 1.21 (0.51 - 2.85) | 1.32 (0.56 - 3.12) | 1.28 (0.54 - 3.04) | 1.27 (0.53 - 3.04) | 1.35 (0.56 - 3.2) |
| History of hypertensive disorder | 0.76 (0.56 - 0.96) | 0.82 (0.67 - 1.01) | 0.77 (0.6 - 0.99) | 0.82 (0.67 - 1.01) | 0.97 (0.61 - 1.57) | 0.9 (0.7 - 1.15) |
| Any high-risk factor-1^st^ pregnancy | Did not converge | 0.95 (0.74 - 1.22) | 1.01 (0.77 - 1.32) | 1.01 (0.71 - 1.42) | 1.01 (0.71 - 1.42) | 1.01 (0.71 - 1.47) |
| Any high-risk factor-2ndpregnancy | 1.03 (0.47 - 2.24) | 1.35 (0.76 - 2.41) | 1.32 (0.75 - 2.36) | 1.34 (0.76 - 2.37) | 1.34 (0.76 - 2.37) | 1.35 (0.73 - 2.52) |
| Renal Disease | 0.68 (0.39 - 1.18) | 0.75 (0.45 - 1.25) | 0.75 (0.45 - 1.24) | 0.75 (0.45 - 1.26) | 0.75 (0.45 - 1.26) | 0.78 (0.4 - 1.54) |
| Diabetes | Did not converge | 0.85 (0.6 - 1.19) | 0.82 (0.58 - 1.16) | 0.85 (0.61 - 1.19) | 0.84 (0.6 - 1.19) | 0.96 (0.65 - 1.43) |
| Chronic hypertension | Did not converge | 1.17 (0.96 - 1.43) | 1.16 (0.95 - 1.42) | 1.17 (0.96 - 1.43) | 1.15 (0.91 - 1.48) | 1.1 (0.86 - 1.42) |
| Multifetal Pregnancy | 1.26 (0.95 - 1.68) | 1.25 (0.96 - 1.64) | 1.31 (0.99 - 1.71) | 1.25 (0.96 - 1.64) | 2.99 (1.2 - 7.44) | 1.21 (0.92 - 1.6) |
| Previous small for gestational age infant | 1.09 (0.83 - 1.42) | 1.17 (0.9 - 1.51) | 1.11 (0.85 - 1.45) | 1.11 (0.85 - 1.45) | 1.17 (0.9 - 1.51) | 0.91(0.61-1.37) |

Table 5. Treatment-covariate interaction coefficients estimated by six methods meta-analysis for the outcome preterm birth <34 weeks and nine covariates

| **Covariate**  **Covariate** | **Meta-analysis of interactions** | **Common interaction effect model one** | **Common interaction effect model two** | **Common interaction effect model three** | **Random interaction effect** | **Within-study model** |
| --- | --- | --- | --- | --- | --- | --- |
|  | OR (95% CI) | OR (95% CI) | OR (95% CI) | OR (95% CI) | OR (95% CI) | OR (95% CI) |
| Family history of hypertensive disorder | 1.08 (0.22 - 5.27) | 1.22 (0.38 - 3.96) | 1.18 (0.39 - 3.59) | 1.23 (0.38 - 3.91) | 1.2 (0.36 - 4.01) | 1.17 (0.37 - 3.68) |
| History of hypertensive disorder | 0.68 (0.5 - 0.93) | 0.74 (0.57 - 0.66) | 0.73 (0.56 - 1.16) | 0.74 (0.57 - 0.97) | 0.93 (0.6 - 1.43) | 0.72 (0.54 - 0.96) |
| Any high-risk factor-1^st^ pregnancy | 1.39 (0.81 - 2.39) | 1.16 (0.86 - 1.56) | 1.14 (0.85 - 1.55) | 1.17 (0.87 - 1.69) | 1.17 (0.87 - 1.57) | 1.13 (0.84 - 1.51) |
| Any high-risk factor-2ndpregnancy | 1.15 (0.68 - 1.96) | 1.06 (0.67 - 1.67) | 1.07 (0.68 - 1.69) | 1.07 (0.68 - 1.69) | 1.07 (0.68 - 1.69) | 0.94 (0.66 - 0.34) |
| Renal Disease | 0.86 (0.44 - 1.67) | 0.87 (0.47 - 1.61) | 0.87 (0.48 - 1.59) | 0.87 (0.47 - 1.6) | 0.87 (0.47 - 1.59) | 1.34 (0.64 - 2.18) |
| Diabetes | 1.34 (0.82 - 2.19) | 1.43 (0.9 - 2.27) | 1.47 (0.92 - 2.38) | 1.42 (0.89 - 2.24) | 1.71 (0.91 - 3.23) | 1.89 (1.11 - 3.23) |
| Chronic hypertension | 0.98 (0.73 - 1.29) | 0.92 (0.72 - 1.19) | 0.93 (0.72 - 1.2) | 0.92 (0.72 - 1.19) | 0.94 (0.67 - 1.29) | 0.94 (0.69 - 1.26) |
| Multifetal Pregnancy | 1.41 (1.02 - 1.95) | 1.33 (1.01 - 1.76) | 1.38 (1.03 - 1.85) | 1.32 (1 - 1.75) | 1.43 (0.98 - 2.09) | 1.33 (1 - 1.77) |
| Previous small for gestational age infant | 0.95 (0.64 - 1.43) | 1.06 (0.74 - 1.51) | 1.04 (0.73 - 1.48) | 1.05 (0.74 - 1.5) | 1.05 (0.74 - 1.5) | 0.80 (0.51-1.25) |

Table 6. Treatment-covariate interaction coefficients estimated by six methods meta-analysis for the outcome small for gestational age infant and nine covariates

| **Covariate**  **Covariate** | **Meta-analysis of interactions** | **Common interaction effect model one** | **Common interaction effect model two** | **Common interaction effect model three** | **Random interaction effect** | **Within-study model** |
| --- | --- | --- | --- | --- | --- | --- |
|  | OR (95% CI) | OR (95% CI) | OR (95% CI) | OR (95% CI) | OR (95% CI) | OR (95% CI) |
| Family history of hypertensive disorder | 1.58 (0.44 - 5.74) | 1.03 (0.32 - 3.37) | 1.21 (0.37 - 4.01) | 1.11 (0.33 - 3.74) | 1.11 (0.33 - 3.71) | 1.22 (0.37 - 4) |
| History of hypertensive disorder | 0.82 (0.54 - 1.23) | 0.84 (0.59 - 1.18) | 0.82 (0.56 - 1.2) | 0.84 (0.58 - 1.21) | 0.84 (0.58 - 1.21) | 0.91 (0.61 - 1.37) |
| Any high-risk factor-1^st^ pregnancy | 1.38 (0.57 - 3.36) | 0.9 (0.64 - 1.27) | 0.89 (0.6 - 1.33) | 0.93 (0.63 - 1.39) | 0.93 (0.63 - 1.38) | 0.92 (0.63 - 1.37) |
| Any high-risk factor-2ndpregnancy | 2.68 (1.02 - 7.05) | 2.09 (1.16 - 3.77) | 2.11 (1.12 - 3.95) | 2.08 (1.14 - 3.83) | 2.08 (1.14 - 3.83) | 2.27 (1.26 - 4.08) |
| Renal Disease | 0.9 (0.26 - 3.1) | 0.96 (0.36 - 2.56) | 0.95 (0.36 - 2.55) | 0.96 (0.36 - 2.55) | 0.96 (0.36 - 2.53) | 1.28 (0.3 - 5.48) |
| Diabetes | 1.02 (0.48 - 2.17) | 1.24 (0.62 - 2.51) | 1.16 (0.55 - 2.44) | 1.14 (0.55 - 2.37) | 1.14 (0.55 - 2.37) | 1.21 (0.55 - 2.65) |
| Chronic hypertension | 0.92 (0.64 - 1.33) | 0.9 (0.66 - 1.23) | 0.87 (0.63 - 1.21) | 0.87 (0.63 - 1.2) | 0.87 (0.63 - 1.2) | 1.02 (0.72 - 1.45) |
| Multifetal Pregnancy | 1.26 (0.81 - 1.99) | 1.48 (1.01 - 2.19) | 1.37 (0.89 - 2.09) | 1.41 (0.92 - 2.15) | 2.38 (1.12 - 5.09) | 1.35 (0.84 - 2.15) |
| Previous small for gestational age infant | 1.04 (0.56 - 1.94) | 1.01 (0.65 - 1.58) | 0.96 (0.61 - 1.54) | 1.01 (0.65 - 1.58) | 1.01 (0.65 - 1.58) | 1.19 (0.62-2.3) |
