# Supplementary figures and images for: Estimating interactions in individual participant data meta-analysis: a comparison of methods in practice
Source: Syst Rev. 2022 Oct 5;11:211. doi: 10.1186/s13643-022-02086-0 (PMC9535994; doi:10.1186/s13643-022-02086-0)

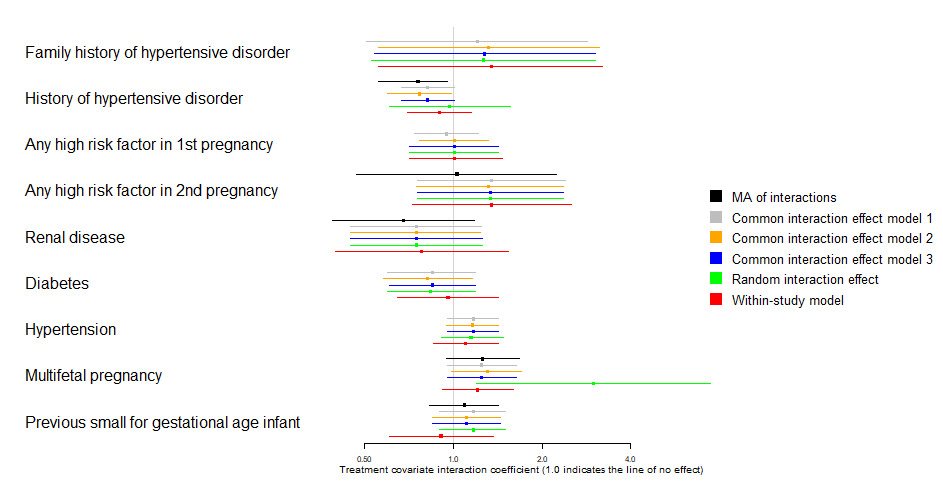

Supplement: Supplementary file 3 — Additional file 3: Supplementary Material Figure 1. Estimates of treatment-covariate interaction for the outcome serious adverse outcome. [file 13643_2022_2086_MOESM3_ESM.tiff]

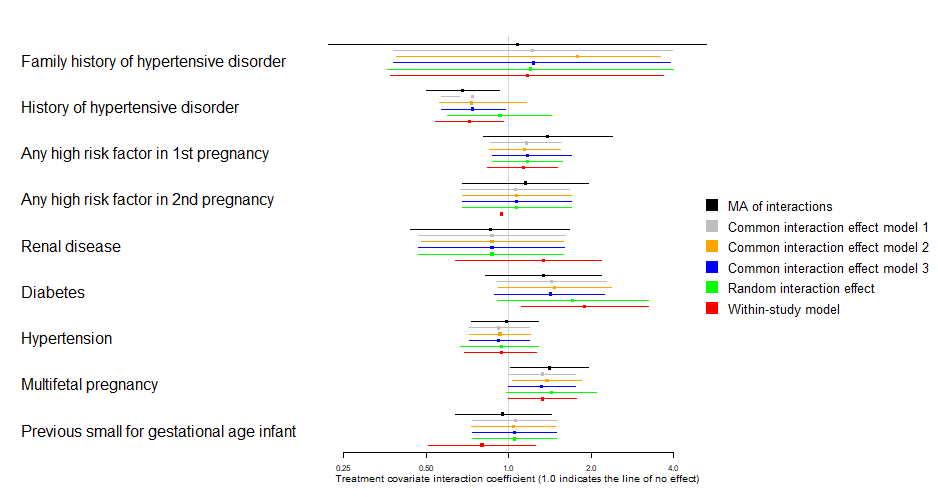

Supplement: Supplementary file 4 — Additional file 4: Supplementary Material Figure 2. Estimates of treatment-covariate interaction for the outcome preterm birth before 34 weeks. [file 13643_2022_2086_MOESM4_ESM.tiff]

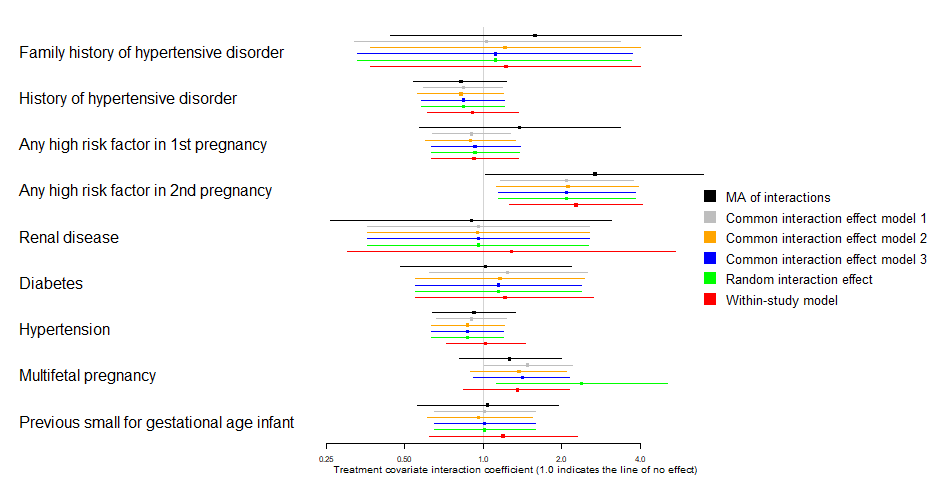

Supplement: Supplementary file 5 — Additional file 5: Supplementary Material Figure 3. Estimates of treatment-covariate interaction for the outcome serious adverse outcome small for. [file 13643_2022_2086_MOESM5_ESM.tiff]
